# Antifungal peptidic compound from the deep-sea bacterium *Aneurinibacillus* sp. YR247

**DOI:** 10.1007/s11274-017-2239-0

**Published:** 2017-03-15

**Authors:** Atsushi Kurata, Yuto Yamaura, Takumi Tanaka, Chiaki Kato, Kaoru Nakasone, Noriaki Kishimoto

**Affiliations:** 1 Faculty of Agriculture, Kindai University, 3327-204 Nakamachi, Nara City, Nara 631-8505 Japan; 2 Department of Marine Biodiversity Research, Japan Agency for Marine-Earth Science and Technology (JAMSTEC), 2-15 Natsushima, Yokosuka, 237-0061 Japan; 3 Faculty of Engineering, Kindai University, 1 Takaya Umenobe, Higashi-Hiroshima City, Hiroshima 739-2116 Japan

**Keywords:** Deep-sea bacterium *Aneurinibacillus* sp. YR247, Antifungal activity, Peptidic compound, *Calyptogena* community, Sagami Bay

## Abstract

*Aneurinibacillus*: sp. YR247 was newly isolated from the deep-sea sediment inside the *Calyptogena* community at a depth of 1171 m in Sagami Bay. The strain exhibited antifungal activity against the filamentous fungus *Aspergillus brasiliensis* NBRC9455. A crude extract prepared from the YR247 cells by ethanol extraction exhibited broad antimicrobial activities. The antifungal compound is stable at 4–70 °C and pH 2.0–12.0. After treatment with proteinase K, the antifungal activity was not detected, indicating that the antifungal compound of strain YR247 is a peptidic compound. Electrospray ionization mass spectrometry of the purified antifungal compound indicated that the peptidic compound has an average molecular weight of 1167.9. The molecular weight of the antifungal compound from strain YR247 is different from those of antimicrobial peptides produced by the related *Aneurinibacillus* and *Bacillus* bacteria. The antifungal peptidic compound from the deep-sea bacterium *Aneurinibacillus* sp. YR247 may be useful as a biocontrol agent.

## Introduction

*Aspergillus* fungi are a common contaminant of bakery and silage products, and some of these fungi produce several mycotoxins (Cheli et al. 2013). Because these compounds can cause economic loss and may affect human health, many approaches have been used to control fungal contamination. Although the approaches are usually based on heat or chemical treatment, antifungal agents produced by microorganisms may alternatively be focused on as biocontrol agents (Igarashi et al. 1997). We previously demonstrated that *Candida maltosa* NP9 from a commercial Iranian cheese generates volatile isoamyl acetate and isoamyl alcohol, which inhibit the spore germination of *Aspergillus brasiliensis* NBRC9455 (Ando et al. 2012, 2015). These volatile compounds also exhibited antifungal activities against various filamentous fungi including *Aspergillus nidulans* NBRC33017, *Aspergillus fumigatus* No.232, *Penicillium chrysogenum* IAM13780, *Rhizopus javanicus* IAM6028, *Mucor roxianus* IAM6131, *Botryotinia fuckeliana* IAM5126, and *Fusarium oxysporum* IAM5009.

*Bacillus* species and related bacteria are industrially useful and produce antimicrobial peptides (Mogi and Kita 2009). *Bacillus subtilis* and *Bacillus amyloliquefaciens* produce iturins (Bonmatin et al. 2003). Iturins are heptapeptides linking the beta-amino fatty acid and iturin A produced by *B. subtilis* RB14 exhibits a strong antibiotic activity with a broad antifungal spectrum (Ohno et al. 1995). *Bacillus brevis* produces gramicidins and tyrocidines (Tang et al. 1992) and *Aneurinibacillus migulanus* ATCC9999^T^ produces gramicidin S (Berditsch et al. 2007b). Gramicidins are pentapeptides (gramicidin A, B, and C) and gramicidin S is a cyclized cationic decapeptide. Tyrocidines is a mixture of cyclic decapeptides (tyrocidine A, B, and C). Gramicidins and Tyrocidines exhibit antimicrobial and antifungal activities. Although gramicidin S has an antibiotic activity against a broad spectrum of Gram-negative and Gram-positive bacteria and several pathogenic fungi, hemolytic activity of gramicidin S restricts its use to topical applications (Mogi and Kita 2009). Under these circumstances, *Bacillus* species and the related bacteria have attracted much attention in the exploration for new antifungal and antibacterial substances.

The exploration of deep-sea environments has emerged as a new frontier in pharmaceutical development (Skropeta 2008). To investigate the utilities of deep-sea microorganisms, we have isolated and characterized several deep-sea microorganisms (Aono et al. 2010; Kurata et al. 2007). Cold seep areas have been found in subduction zones of continental margins, mud volcanoes, and deep-sea trenches (Kulm et al. 1986; Li et al. 1999a, b), and the microbial diversity in cold-seep biological communities has received attention because of those specific microbial processes (Heijs et al. 2007; Sibuet and Olu 1998). The biological communities in the cold seep areas consist of mussels, clams, and tubeworms, and are supported by microbial chemosynthesis utilizing methane, sulphur, sulphide, and so on, seeping through the geological processes of the seafloor (Boetius et al. 2000; Fujiwara et al. 2001; Li and Kato 1999). Study of the microbial diversity of the *Calyptogena* sediment (depth; 1168 m) and bacterial mat (depth; 1174 m) in Sagami Bay revealed that the microbial community at this site is distinct from other cold seep sites (Fang et al. 2006). *Epsilonproteobacteria*, which are abundant in other cold seep sites and hydrothermal vents (Urakawa et al. 1999, 2000), are absent from the cold-seep area of Sagami Bay. The *Calyptogena* microbial community in the cold seep area of Sagami Bay consists mainly of bacteria that be closely related to *Shewanella* sp. (*Gammaproteobacteria*) and *Desulfobulbus* sp. (*Deltaproteobacteria*) and some archaea that be closely related to *Sulfolobus* sp. (*Crenarchaeota*) and *Methanococcus* sp. (*Euryarchaeota*). Although a variety of bacteria and archaea have been generally elucidated in those cold seep biological communities, very little is known concerning microorganisms that exhibit antimicrobial activities.

In this study using a deep-sea sediment (depth; 1171 m) of the *Calyptogena* community of Sagami Bay as the isolation source, we explored the microorganism that produces an antifungal substance against filamentous *A. brasiliensis*. The bacterial isolate was phylogenetically characterized, and its inhibitory activity against fungus, yeast, and Gram-positive and Gram-negative bacteria was evaluated. Additionally, the properties of the antifungal compound were analyzed.

## Materials and methods

### Isolation of the deep-sea bacterium with antifungal activity

A deep-sea sediment inside the *Calyptogena* community was collected using Hyper-Dolphin remotely operated vehicle in the Sagami Bay (35°0.072′N 139°13.477′E), Japan, at a depth of 1171 m in January 2010 (Hiyoshi et al. 2011). The deep-sea sediment was collected and immediately stored at −80 °C. After 7 months, bacterial isolation was carried out. The deep-sea sediment is fine-grained mud and an aliquot of the sample was spread on a modified LB plate consisting of (w/v) 0.1% Bacto tryptone (Difco), 0.5% Bacto yeast extract (Difco), 0.5% glucose, 1.0% NaCl, 0.2% MgSO_4_·7H_2_O, 0.02% K_2_HPO_4_, and 1.5% agar (pH 7.4). After incubation at 37 °C for 2–3 days, several colonies had grown on the plate. For detection of the antifungal activity against the filamentous fungus *A. brasiliensis* NBRC9455, the colonies were inoculated onto a spore-mixed LB modified plate (pH 7.4) consisting 1.0 × 10^4^ spores/ml *A. brasiliensis* NBRC9455 spores. After incubation at 37 °C for 3 days, colonies with inhibition zones of *A. brasiliensis* NBRC9455 were isolated as antifungal substance-producing microorganisms. The colonies were repeatedly purified and separated, suspended in a 15% glycerol, and stored at −80 °C.

### Phylogenetic analysis of bacterial isolate

The bacterial isolate YR247, which exhibits high antifungal activity against (*A*) *brasiliensis* NBRC9455, was evaluated. To determine phylogenetic relationships, 1509 bp of the 16S rRNA gene fragment was amplified from DNA fraction of strain YR247 by PCR. DNA fraction was extracted from bacterial cell pellet of strain YR247 using an Illustra bacteria genomic Prep Mini Spin Kit (GE Healthcare, Munich, Germany). The genomic DNA of strain YR247 was used as a template. The 16S ribosomal DNA sequence was amplified and sequenced using universal primers 27f: 5′-GAGTTTGATCCTGGCTCAG-3′ and 1525r: 5′-AAAGGAGGTGATCCAGCC-3′ (Lane 1991). The reaction solution was 50 µl, including 0.5 µl Blend Taq (2.5 U/µl, Toyobo Co. Ltd., Osaka, Japan), 5 µl 10× PCR Buffer for Blend Taq, 5 µl dNTPs (2 mM), 20 ng template DNA, 1 µl 27 f primer (10 µM), 1 µl 1525r primer (10 µM) and 36.5 µl ddH_2_O. The amplification was conducted by polymerase chain reaction in a PCR thermal cycler (MyCycler, Bio-Rad Laboratories Inc., Richmond, CA). The program for the PCR was as follows: denaturation at 94 °C for 7 min followed by 30 cycles of denaturation at 94 °C for 45 s, annealing at 55 °C for 45 s, and extension at 72 °C for 45 s. The DNA sequencing was carried out by FASMAC DNA Sequencing Services (Kanagawa, Japan). Nucleotide substitution rates (*K*nuc) were determined, and a distance matrix tree was constructed by using the neighbor-joining method with the CLUSTAL_X program (Kimura 1980; Saitou and Nei 1987; Thompson et al. 1997). Alignment gaps and unidentified base positions were not taken into consideration for the calculations. The topology of the phylogenetic tree was evaluated by performing a bootstrap analysis with 1000 replications (Felsenstein 1985). NJ plot software (http://pbil.univ-lyon1.fr/software/njplot.html) was used to prepare a graphical view of the phylogenetic tree. The 16S rRNA gene sequence of (*B*) *subtilis* IAM12118^T^ (AB042061) was used as an out group.

### Production and purification of the antifungal compound

Strain YR247 was aerobically cultured in 2.4 l of LB modified media containing 0.1% Bacto tryptone (Difco), 0.5% Bacto yeast extract (Difco), 0.5% glucose, 1.0% NaCl, 0.2% MgSO_4_·7H_2_O, and 0.02% K_2_HPO_4_ (pH 7.4) at 37 °C for 24 h. After cultivation, a 13.5 g cell pellet was obtained by the centrifugation at 6000×*g* at 25 °C for 10 min. The crude extract was prepared according to the reference (Nakai et al. 2005). The cell pellet was resuspended in 225 ml of ethanol, and the cell suspension was then heated at 95 °C for 3.0 min. After centrifugation of the heated cell suspension at 6000×*g* at 25 °C for 10 min, 220 ml of the supernatant containing the antifungal compound was collected and placed on a rotary evaporator. The solvent was removed at 37 °C and 30 hPa for 30 min, and 150 mg of the residue obtained was resuspended in methanol and filtered through a 0.2 µM filter to prepare the 39 mg/ml crude extract.

The solvent of the 39 mg/ml crude extract containing the residue (150 mg) was changed to ethyl acetate. Then, the crude extract in ethyl acetate was submitted to silica gel chromatography (C200, Wako Pure Chemical, Osaka, Japan, 1.2 × 20 cm). Elution was performed using ethyl acetate, ethyl acetate:2-propanol (1:1, v/v), and methanol consecutively. Using agar-spot assay and ninhydrin coloration, active fractions were determined and collected. The residue in the active fractions was obtained by rotary evaporation at 37 °C and 30 hPa for 30 min. The residue (30 mg) was dissolved in ethanol:2-propanol (1:1, v/v) and subjected to silica gel chromatography (C200, Wako Pure Chemical, Osaka, Japan, 1.2 × 20 cm). Elution was performed using ethanol:2-propanol (1:1, v/v), and the active fraction was collected. The residue (15 mg) was concentrated from the active fraction by the rotary evaporation and resuspended in methanol. The methanol fraction was subjected to preparative thin-layer chromatography. The residue in the methanol fraction was developed on thin-layer chromatography (TLC) silica gel plates (TLC aluminum sheets, 8 × 8 cm, SilicaGel 60F254, Merck Co. USA), followed by detection of the antifungal compound by TLC bioautography overlay assay and ninhydrin reaction. The active spot area of another developed TLC plate was collected, and the antifungal compound was extracted with an aliquot of methanol. The methanol fraction containing the residue (2.3 mg) was used as the purified antifungal compound.

### Detection of antimicrobial activity

Antifungal activity against *A. brasiliensis* NBRC9455 was determined by agar-spot and TLC bioautography overlay assays. For the agar-spot assay, 50 µl of the crude extract at 5.0 mg/ml-methanol from strain YR247 was spotted onto the surface of the spore-mixed LB modified plate (pH 7.4) consisting of 1.0 × 10^4^ spores/ml *A. brasiliensis* NBRC9455 spores. After incubation for 3 days at 37 °C, the inhibition zone (mm) at the spotted site was measured. For the TLC bioautography overlay assay, 10 µl of the purified antifungal compound at 1.0 mg/ml was developed using acetone:distilled water (7:1, v/v) on a TLC silica gel plate (TLC aluminum sheets, 8 × 8 cm, SilicaGel 60F254, Merck Co. USA). The developed TLC plate was placed in a petri dish and covered with the spore-mixed LB modified plate (pH 7.4) containing 1.0 × 10^4^ spores/ml *A. brasiliensis* NBRC9455 spores. After incubation for 3 days at 37 °C, the inhibition zone of the developed plate was detected. Using another developed TLC plate, the antifungal compound was detected by ninhydrin reaction. Antimicrobial activities against *C. albicans* NBRC1594, *S. aureus* NBRC12732^T^, and *E. coli* NBRC3301^T^ were evaluated by the agar-spot assay described above. The crude extract (50 µl at 5 mg/ml-methanol) was spotted on each plate containing 1.0 × 10^7^ cfu/ml of each microorganism cells. After incubation 1–2 days at 28–37 °C, the inhibition zone on the spotted place was checked. All experiments for evaluation of the antimicrobial activity were carried out in triplicate.

### Effects of temperature, pH, and proteinase K on antifungal activity

To examine the effect of temperature, the crude extract (5 mg/ml) was incubated at 4, 10, 28, 37, 60, 70, 80, 90, and 100 °C for 6 h respectively. After the sample was cooled to room temperature, the residual antifungal activity of the treated sample (50 µl) was tested by agar-spot assay using *A. brasiliensis* NBRC9455.

To evaluate the pH stability of the purified antifungal compound, pH values of the crude extract (5.0 mg/ml) were adjusted from 2.0 to 12.0 at intervals of 2.0 by substitution of each solvent by ultrafiltration, and the adjusted crude extract kept at 4 °C for 12 h. Afterward, the pH values were readjusted to 7.0, and the residual antifungal activity of the treated sample (50 µl at the final concentration of 5.0 mg/ml-distilled water) was tested by the agar-spot assay using *A. brasiliensis* NBRC9455.

For evaluation of the effect of proteinase K, the crude extract (5.0 mg/ml-distilled water) was treated with 1.7 mg of proteinase K (56 unit, Nacalai Tesque, Inc., Kyoto, Japan) at 37 °C for 30 min. The residual antifungal activity of the treated sample (50 µl) was tested by an agar-spot assay using *A. brasiliensis* NBRC9455. As positive and negative controls, the crude extract not treated with proteinase K and proteinase K solution not containing the crude extract were used, respectively, in the agar-spot assay.

### Analysis of the antifungal compound by mass spectrometry

Electrospray ionization mass spectrometry (ESI–MS) analysis was performed to determine the molecular weight of the antifungal compound. The purified antifungal compound (50.0 µg/ml) was introduced into a mass spectrometer (Waters Q-TOF Premier, Micromass MS Technologies, Manchester, UK) at a flow rate of 10.0 μl/min with the capillary voltage of 2.5 V, collision energy of 5.0 V, and source temperature of 100 °C.

## Results

### Isolation and identification of *Aneurinibacillus* sp. YR247

**Fig. 1:**
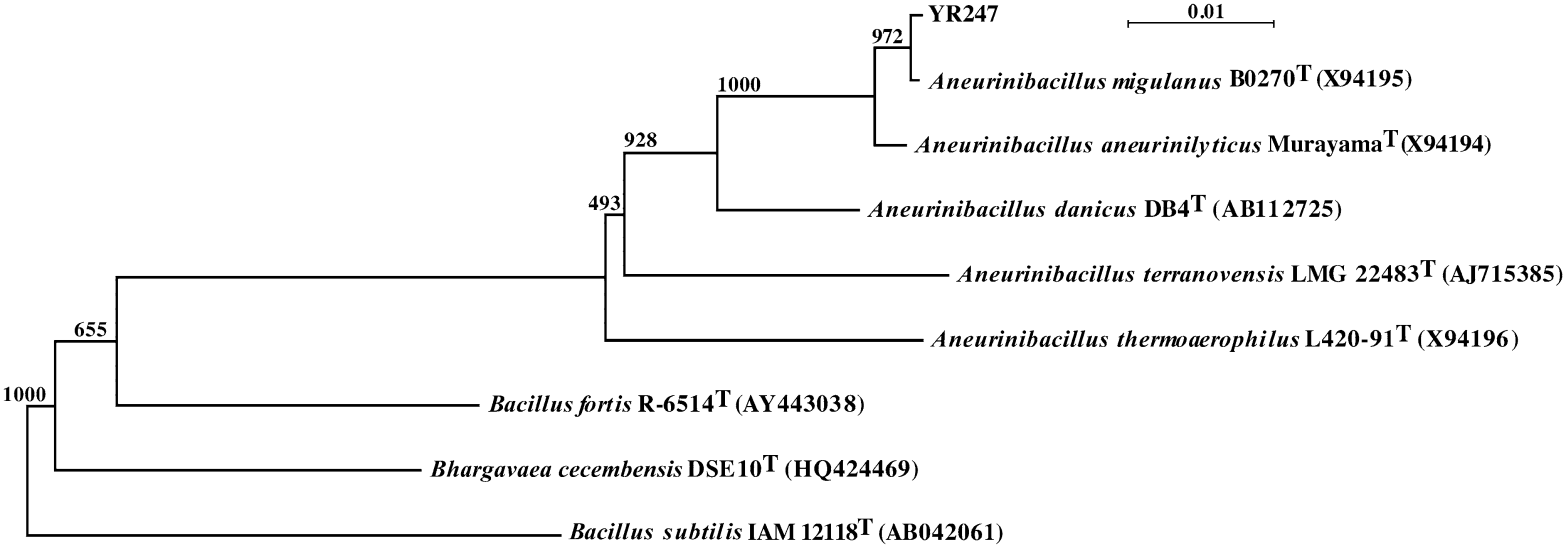
Phylogenetic tree showing the relationships between strain YR247 and related bacteria based on 16S rRNA gene sequences. Bootstrap values were calculated from multiple resamplings of the sequence data set, which are the basis for multiple tree topologies. GenBank accession numbers of 16S rRNA gene sequences are given in parentheses. *Bar* 0.01 nucleotide substitutions per site

To establish an effective microorganism for the control of fungal contamination, antagonistic bacteria were isolated from the deep-sea sediment taken at Sagami Bay, Japan, at a depth of 1171 m. One of them, strain YR247, exhibited a distinct antifungal activity against *A. brasiliensis* NBRC9455. Its 16S rRNA sequence consists of 1509 nucleotides. The results of phylogenetic analysis based on 16S rRNA gene sequence data are shown in Fig. 1; strain YR-247 clearly falls within the genus *Aneurinibacillus* and belongs to *Firmicutes*. The nucleotide sequence of the 16S rRNA gene of strain YR-247 was most closely related to those of *Aneurinibacillus migulanus* B0270^T^ (X94195) and *Aneurinibacillus aneurinilyticus* Murayama^T^ (X94194) with similarities of 99% for both. The next closest level of similarity was *Aneurinibacillus danicus* DB4^T^ (AB112725) with 97% identity. The 16S rRNA sequence of strain YR247 was submitted to NCBI GenBank (accession no. LC110197).

### Antimicrobial activity of the crude extract

Although we tried the detection of antifungal activity using culture supernatant of strain YR247, the antifungal activity against *A. brasiliensis* NBRC9455 was not detected (data not shown). Therefore, we prepared the crude extract from the bacterial cell pellet and examined the antifungal activity. As the results, the antifungal activity of the crude extract against *A. brasiliensis* NBRC9455 was detected. As shown in Table 1, the crude extract exhibited a wide antimicrobial activity against fungus *A. brasiliensis* NBRC9455, Gram-positive bacterium *S. aureus* NBRC12732^T^ and Gram-negative bacterium *E. coli* NBRC3301^T^, but not yeast *C. albicans* NBRC1594. The inhibitory zones remained the same for several days (data not shown). Antimicrobial activities by the solvent against test strains were not detected by the agar spot assay.

**Table 1:** Antimicrobial activities of the crude extract from *Aneurinibacillus* sp. YR247 cells

Test strains	Inhibitory activity	Inhibition zone (mm)
*Aspergillus brasiliensis*	+	20.3
*Candida albicans*	−	0.0
*Staphylococcus aureus*	+	21.7
*Escherichia coli*	+	14.9

Using 50 µl of the crude extract (5 mg/ml-methanol), an agar-spot assay was carried out in triplicate

+ inhibited, − not inhibited

### Effect of temperature, pH, and proteinase K on antifungal activity

**Fig. 2:**
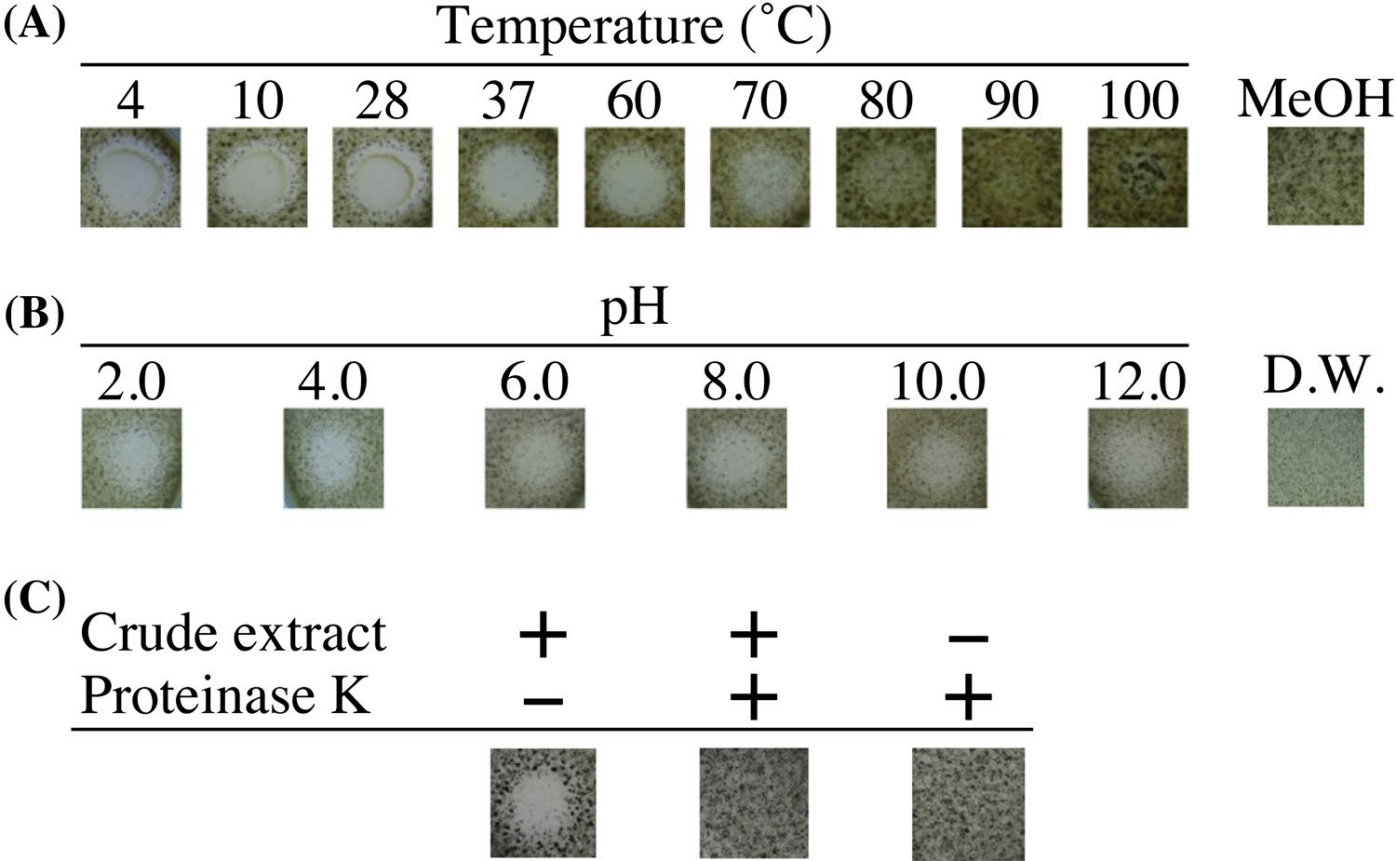
Effects of temperature, pH, and proteinase K on the antifungal compound. Using 5 mg/ml of the crude extract prepared from YR247 cells, the residual antifungal activities were evaluated by agar-spot assay in triplicate. After incubation at each temperature (**a**), each pH (**b**), and with or without proteinase K (**c**), each residual antifungal activity was evaluated. + addition, − no addition. *MeOH* methanol, *D.W*. distilled water

To examine the thermostability, the crude extract containing the antifungal compound was incubated at different temperatures for 6 h. The residual antifungal activity was tested by an agar-spot assay (Fig. 2a). Treatment at 4–70 °C did not decrease the antifungal activities (inhibition zone: 19.0–20.3 mM), whereas the activity was only slightly detected after being treated at 80 °C. The activities were not detected when the crude extracts were treated at 90 and 100 °C. As shown in Fig. 2b, the antifungal activity was not affected at pH 2.0–12.0 after incubation at 4 °C for 12 h (inhibition zone: 13.5–16.3 mM). Although antifungal activity was detected without the proteinase K treatment (inhibition zone: 13.5 mM), the antifungal activity was not observed after being treated with proteinase K at 37 °C for 30 min (Fig. 2c).

### Purification and mass analysis of the antifungal compound

**Fig. 3:**
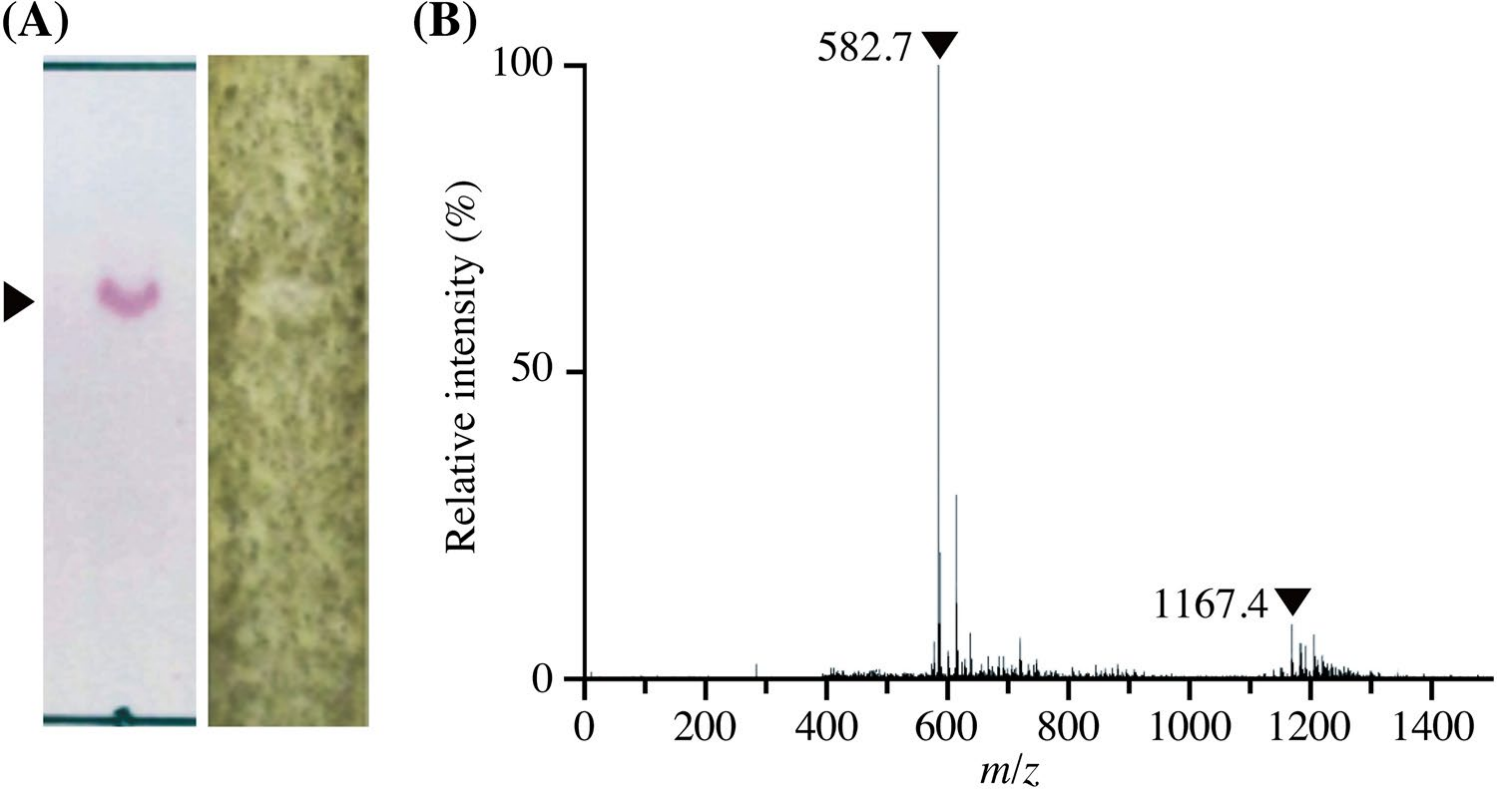
TLC bioautography overlay assay and ESI–MS analysis of purified antifungal compound. A TLC bioautography overlay assay was carried out using 10 µl of the 1.0 mg/ml purified antifungal compound. **a** The purified antifungal compound (R_f_ value; 0.62) is indicated by *triangles*. Detections by ninhydrin reaction (*left*) and TLC bioautography overlay assay (*right*) are indicated. The detection of antifungal compound was carried out in triplicate. **b** ESI–MS analysis of the 50 µg/ml purified antifungal compound indicated the doubly charged ion peak (*m/z* 1167.4) and the charged ion peak (*m/z* 582.7)

Because the crude extract obtained from the YR247 cells stably exerted antifungal activity, it was further purified using silica gel chromatography and preparative thin-layer chromatography. Approximately 2.3 mg of the antifungal compound was purified from the YR245 cell pellet (13.5 g). The R_f_ value of the antifungal compounds was 0.62 using acetone:distilled water (7:1, v/v) on TLC silica gel plates (Fig. 3a). As shown in the mass spectrum at negative ion mode of the purified fraction (Fig. 3b), the molecular weight of the antifungal compound was estimated to be 1167.9 because the molecular ion peaks at *m/z* 1167.4 and 582.7 probably correspond to the charged ion [M−H]^−^ and the doubly charged ion [M−2H]^2−^, respectively (Gibson et al. 1993; Tilvi and Naik 2007). The calculation of molecular weight is indicated as follows:

$$[{\text{M}} - {\text{H}}];\,{\text{1167}}{\text{.4 + 1}}{\text{.0 = 1168}}{\text{.4}}$$

$$[{\text{M}} - 2{\text{H}}];\,582.7 \times 2 + 2.0 = 1167.4$$

The molecular weight of the antifungal compound was estimated as 1167.9 using the average of both values (1168.4 and 1167.4).

## Discussion

*Aneurinibacillus* sp. YR247 was isolated from the deep-sea sediment inside the *Calyptogena* community at a depth of 1171 m in Sagami Bay of Japan. The strain exhibited antifungal activity against *A. brasiliensis* NBRC9455. Using the crude extract prepared from YR247 cells, the antimicrobial activities were detected against *A. brasiliensis* NBRC9455, Gram-positive bacterium *S. aureus* NBRC12732^T^, and Gram-negative bacterium *E. coli* NBRC3301^T^, but not yeast *C. albicans* NBRC1594. The antifungal compound from strain YR247 was stable at 4–70 °C and pH 2.0–12.0. The antifungal compound was shown to have a rather stable structure as it retained activity after incubation at high temperatures and over a wide pH range as well as bacteriocin-like inhibitory substance from *Bacillus cereus* ATCC 14,579 and antifungal peptides from *Bacillus* sp. BH072 (Risøen et al. 2004; Zhao et al. 2013). After treatment with proteinase K, the antifungal activity was not detected, indicating that the antifungal compound is a peptidic compound (Fig. 2c).

Among the related strains, it was already reported that *Aneurinibacillus migulanus* ATCC9999^T^ produces the cyclic peptide gramicidin S (Berditsch et al. 2007a). *Bacillus brevis* produces a mixture containing linear peptides, namely, gramicidin A (valine-gramicidin A and isoleucine-gramicidin A), gramicidin B (valine-gramicidin B and isoleucine-gramicidin B), and gramicidin C (valine-gramicidin C and isoleucine-gramicidin C) (Tang et al. 1992). *B. brevis* also produces cyclic peptides, namely, tyrocidine A, tyrocidine B, and tyrocidine C. It was also reported that those peptides exhibit antimicrobial activities against Gram-positive bacteria including *S. aureus* and *B. subtilis* and Gram-negative bacteria including *E. coli* (Danders et al. 1982). Additionally, iturin A-like peptides from *Bacillus* sp. BH072 exhibited antifungal activities against *A. brasiliensis* (previously called (*A*) *niger*), *Pythium*, and *Botrytis cinerea* and had no inhibition on *E. coli* and *S. aureus* (Zhao et al. 2013). Fengycin-like peptide and iturin-like peptides from *Bacillus amyloliquefaciens* LBM 5006 exhibited antifungal activities against *Aspergillus* spp., *Fusarium* spp., and *Bipolaris sorokiniana* (Benitez et al. 2010). Bacillomycin-like peptides from (*B*) *subtilis* B-FS06 inhibited the growth and spore germination of *Aspergillus flavus* (Zhang et al. 2008). Compared with related studies, we indicated that the antifungal peptide from strain YR247 is responsible for the anti-*Aspergillus* activity.

ESI–MS analysis of the antifungal peptide from strain YR247 indicated an average molecular weight of 1167.9 (Fig. 3). The molecular weights of gramicidin S (MW 1140.5), valine-gramicidin A (MW 1881.1), isoleucine-gramicidin A (MW 1895.1), valine-gramicidin B (MW 1842.1), isoleucine-gramicidin B (MW 1856.1), valine-gramicidin C (MW 1858.1), isoleucine-gramicidin C (MW 1872.1), tyrocidine A (MW 1269.7), tyrocidine B (MW 1308.7), tyrocidine C (MW 1347.7), iturin A-like peptides (MW 1057.6, 1071.5, 1016.7, 1030.5, 1044.8, 1034.7, and 1058.9), fengycin-like peptide (MW 1464), and bacillomycin-like peptides (MW 1071.6) have been reported (Benitez et al. 2010; Laiko et al. 2000; Tang et al. 1992; Zhang et al. 2008; Zhao et al. 2013). Comparison of each molecular weight indicated that the antifungal compound from strain YR247 (MW 1167.9) is structurally different from these antimicrobial peptides. Based on these results, the deep-sea bacterium *Aneurinibacillus* sp. YR247 probably produces a novel antifungal peptide.

In the case of strain YR247, Table 1 suggests that the antifungal peptidic compound exhibits the similar bactericidal activities. In addition to these bactericidal activities, gramicidin S from *A. migulanus* ATCC9999^T^ exhibits antifungal activity against *C. albicans* (Kondejewski et al. 1996). However, in the case of strain YR247, although antifungal activity against *A. brasiliensis* NBRC9455 was detected, antifungal activity against *C. albicans* NBRC1594 was not detected (Table 1). Thus, the results suggest that the peptidic compound from strain YR247 is distinct from gramicidin S. The antifungal activity of iturin and related lipopeptides depends on the composition of the length of the lipid chain (Maget-Dana and Peypoux 1994; Moyne et al. 2001). We indicated antifungal peptide with molecular weight of 1167.9. We suggested that two methylene groups (–CH_2_–) in the aliphatic chain of the antifungal peptide is more than that of gramicidin S (MW 1140.5). The difference in antimicrobial activity against *C. albicans* may depends on the composition of the length of the aliphatic chain.

Action mechanism of antibacterial activity by gramicidin S is assumed to be the accumulation of significant amounts of gramicidin S in a membrane, resulting in the destruction of the membrane integrity and a leakage of alkaline metal cations and protons (Mogi and Kita 2009). Although gramicidin S seems to be useful as a biocontrol agent against the agricultural pathogens, the hemolytic activity of gramicidin S restricts its use to topical applications. In this study, we purified gramicidin-like novel antifungal peptide from the deep-sea bacterium. The hemolytic activity of the antifungal peptide from strain YR247 should be examined and antibacterial and antifungal spectra needs to be determined. We expect elucidation of the action mechanism of the antifungal peptide from YR247 is useful for development of the biocontrol agent against the food and agricultural pathogens.
